# A Bombesin-Shepherdin Radioconjugate Designed for Combined Extra- and Intracellular Targeting

**DOI:** 10.3390/ph7060662

**Published:** 2014-05-27

**Authors:** Christiane A. Fischer, Sandra Vomstein, Thomas L. Mindt

**Affiliations:** University of Basel Hospital, Clinic of Radiology and Nuclear Medicine, Division of Radiopharmaceutical Chemistry, Petersgraben 4, 4031 Basel, Switzerland; E-Mails: christiane.fischer@usb.ch (C.A.F.); Sandra.Vomstein@usb.ch (S.V.)

**Keywords:** multifunctional radioconjugates, intra- and extracellular targeting, tumor-targeting, ^99m^Tc-tricarbonyl, bombesin, shepherdin, gastrin releasing peptide receptor, Hsp90, SPECT

## Abstract

Radiolabeled peptides which target tumor-specific membrane structures of cancer cells represent a promising class of targeted radiopharmaceuticals for the diagnosis and therapy of cancer. A potential drawback of a number of reported radiopeptides is the rapid washout of a substantial fraction of the initially delivered radioactivity from cancer cells and tumors. This renders the initial targeting effort in part futile and results in a lower imaging quality and efficacy of the radiotracer than achievable. We are investigating the combination of internalizing radiopeptides with molecular entities specific for an intracellular target. By enabling intracellular interactions of the radioconjugate, we aim at reducing/decelerating the externalization of radioactivity from cancer cells. Using the “click-to-chelate” approach, the ^99m^Tc-tricarbonyl core as a reporter probe for single-photon emission computed tomography (SPECT) was combined with the binding sequence of bombesin for extracellular targeting of the gastrin-releasing peptide receptor (GRP-r) and peptidic inhibitors of the cytosolic heat shock 90 protein (Hsp90) for intracellular targeting. Receptor-specific uptake of the multifunctional radioconjugate could be confirmed, however, the cellular washout of radioactivity was not improved. We assume that either endosomal trapping or lysosomal degradation of the radioconjugate is accountable for these observations.

## 1. Introduction

**Figure 1:**
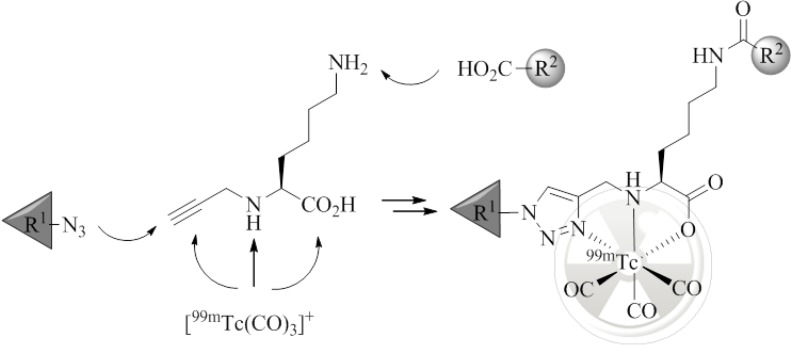
Assembly of multifunctional, ^99m^Tc-tricarbonyl-labeled peptide conjugates by amide bond formation, CuAAC, and (radio)metal complexation; residues R^1^ and R^2^ represent two different entities of biological function (e.g., derivatives of BBS and shepherdin) to be combined in the final radioconjugate.

Regulatory peptides are known to display high specificity and affinity towards different G-protein coupled receptors (GPCRs) which are overexpressed on the cell membrane of various cancer cells [1]. On this account, a number of radiopharmaceuticals based on these peptides as tumor-targeting vectors are currently under preclinical and clinical evaluation or have already found application in nuclear medicine for the management of cancer [2,3,4]. However, after receptor-mediated uptake of radiolabeled regulatory peptides into tumors, a rapid washout of a significant fraction of radioactivity is often observed [5,6,7,8,9]. This not only renders the initial targeting efforts in part futile but may also impair imaging quality and the efficacy of radiopharmaceuticals where therapeutic radionuclides are employed. To overcome these limitations, new strategies are needed to enhance the cellular retention of radioactivity inside cancer cells and tumors. A possible approach to achieve this goal is represented by the application of radiolabeled conjugates which combine extra- and intracellular targeting. Such multifunctional radioconjugates have the promise to be recognized first by an extracellular target (e.g., a GPCR) that triggers cell internalization by endocytosis. Once inside the cell, the radioconjugate interacts with its intracellular target (e.g., an organelle or protein) by means of which the radioactive cargo is trapped [10]. Radioconjugates consisting of two molecular moieties, each of which specific for an extra- and intracellular target, respectively, have been reported; however, examples remain scarce. Ginj *et al.* have reported the combination of radiolabeled somatostatin derivatives with a nuclear localization sequence (NLS) to transport Auger electron emitting radionuclides to the cell nucleus. The reported conjugates target specifically the cell nucleus and display a decreased externalization rate *in vitro*; however, no *in vivo* data is available [6]. With the same goal, the groups of Alberto and Santos have combined a ^99m^Tc-labeled bombesin (BBS) derivative with the DNA intercalator acridine orange, which simultaneously serves as a fluorescent probe for optical imaging. The *in vitro* results reported on the ability of the conjugates to target the cell nucleus are not consistent and data on the externalization of radioactivity from cells is not reported [11,12]. The Garrison group investigated the combination of a radiolabeled BBS derivative with 2-nitroimidazoles, a hypoxia-specific prodrug. Upon enzymatic reduction of the 2-nitroimidazole moiety, the radioconjugate gets covalently linked to intracellular proteins. While an enhancement of the retention of radioactivity in PC-3 cells as a result of 2-nitroimidazole moieties was demonstrated under hypoxic conditions *in vitro*, the effect was less pronounced in a mouse model [13]. Finally, we have recently reported a ^99m^Tc-tricarbonyl-labeled, dual-targeting radiopeptide conjugate made of a modified amino acid sequence of BBS, [Nle^14^]BBS(7-14) (QWAVGHLNle), for extracellular targeting of the gastrin-releasing peptide receptor (GRP-r) and a triphenylphosphonium (TPP) entity specific for mitochondria by its ability to accumulate electrophoretically driven in the energized membrane of the organelle [10]. While receptor-specific cell internalization of the conjugate could be confirmed, the cellular washout of radioactivity was not reduced. We tentatively ascribed our observations to the possibility of hindered passage of the TPP moiety through the membrane of mitochondria because of the peptide it is attached to; similar observations have been reported for cell penetrating peptide/TPP conjugates [14]. We therefore set out to investigate the utility of multifunctional radiopeptides which target cytosolic proteins and thus, do not require multiple passages through intracellular membranes, a process considered as a major barrier for targeting intracellular epitopes [15]. With these considerations in mind, we chose the cytosolic chaperone, heat shock protein 90 (Hsp90), as a potential intracellular target for our purpose. Hsp90 is a ubiquitous protein important for, e.g., cell proliferation and survival. As such, it is found overexpressed by nearly all cancer cells in high concentrations [16,17,18]. Hsp90 assists and controls the non-covalent folding/unfolding of many client proteins, including the peptide survivin [17,19,20]. The group of Altieri has identified the binding sequence of survivin(K79-L87), termed “shepherdin” as an inhibitor of the survivin-Hsp90 interaction and Hsp90 ATPase activity [21]. The high affinity and specificity towards Hsp90 and anticancer activities of shepherdin(79–87) (KHSSGCAFL) and its truncated form shepherdin(79–83) (KHSSG), respectively, have been demonstrated by different approaches [20,21,22,23,24]. However, applications of radiolabeled derivatives of shepherdin or its combination with tumor-targeting peptides for receptor-specific delivery have not yet been described. Herein, we wish to report the synthesis of BBS-shepherdin radioconjugates prepared by the previously reported modular “click-to-chelate” approach [25,26] (Figure 1) and their evaluation *in vitro*.

## 2. Experimental Section

### 2.1. General Procedures

General procedures, solvents, chemicals, synthesis equipment, analytic instruments (HPLC, MS, NMR, gamma counter) were previously described [10,27]. The “CRS Isolink kit” (Center for Radiopharmaceutical Science, Paul Scherrer Institute, Villigen, Switzerland) was used for the preparation of the precursor [^99m^Tc(CO)_3_(H_2_O)_3_]^+^. For analytical separation, a C12 reversed-phase column Phenomenex Jupiter 4u Proteo 90 Å, 4 µm, 250 × 4.6 mm (column A) was used and preparative purification was performed on a Macherey Nagel Nucleodur C18 ISIS, 5 µm, 250 × 16 mm (column B). HPLC solvents were 0.1% trifluoroacetic acid (TFA) in water (A) and 0.1% TFA in acetonitrile (B). Quality control of (radio) metal-labeled peptides was performed using column A and a linear gradient from 80% A to 50% A in 20 min with a flow rate of 1.5 mL/min, or a linear gradient from 80% A to 60% A in 20 min. Peptide purification was performed using column B and different linear gradients with a flow rate of up to 8 mL/min. The observed *m/z* correspond to the monoisotopic ions. Chemical shifts are reported in parts per million (ppm) and coupling constants (*J*) in Hertz (Hz) from high to low field. Standard abbreviations indicating multiplicity are singlet (s), doublet (d), doublet of doublets (dd), triplet (t), and multiplet (m).

### 2.2. Synthetic Procedures

#### 2.2.1. Organic Synthesis

**Scheme 1:**
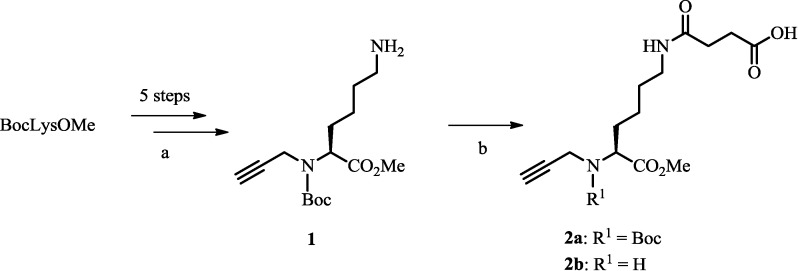
Synthesis of central lysine-based precursor. (a) see reference [28]; (b) succinic anhydride, *i*-Pr_2_NEt, CH_2_Cl_2_, 2 h, rt.

*N*(α)Boc-*N*(α)propargyl-Lys(OMe) **1** was synthesized in five steps from commercial BocLysOMe according to published procedures [28]. Subsequently, succinic anhydride (2 equiv. 2.22 mmol) was coupled to *N*(α)Boc-*N*(α)propargyl-Lys(OMe)*HCl (**1**, 370 mg, 1.11 mmol) *via* amide bond formation in CH_2_Cl_2_ under basic conditions (*i*-Pr_2_NEt; 6 equiv.) for two hours at rt (Scheme 1). The reaction mixture was concentrated under reduced pressure and the crude product was dissolved in ethyl acetate. The organic phase was extracted with citric acid (0.1 M) and saturated sodium hydrogen carbonate. The organic layer was disposed. The hydrogen carbonate phase was acidified with hydrogen chloride (5%) under stirring and extracted twice with fresh ethyl acetate. The organic phases were combined, dried over magnesium sulfate, and concentrated under reduced pressure to yield compound **2a** as a pale yellowish oil (435.3 mg, 91%; see Scheme 1). For characterization purposes, the Boc protecting group of **2a** was removed (CH_2_Cl_2_/TFA, 2:1; 5 h; rt) and the product was analyzed as its TFA salt **2b**; ^1^H-NMR (400 MHz, MeOH-d_4_; recorded after completed H/D exchange): δ = 4.10 (dd, 1H, *J* = 7.0 Hz, *J* = 5.0 Hz), 3.96 (dd, 1H, *J* = 16.6 Hz, *J* = 2.5 Hz), 3.92 (dd, 1H, *J* = 16.6 Hz, *J* = 2.5 Hz), 3.79 (s, 3H), 3.18 (t, 1H, *J* = 2.5 Hz), 3.12 (m, 2H), 2.52 (t, 2H, *J* = 6.9 Hz), 2.38 (t, 2H, *J* = 6.9 Hz), 1.97–1.85 (m, 2H), 1.51–1.44 (m, 2H), 1.43–1.25 (m, 2H) ppm; ^13^C-NMR (MeOH-d_4_): δ = 176.42, 174.81, 170.19, 162.22 (TFA, q, *J*_C-F_: 35.7 Hz), 117.86 (TFA, q, *J*_C-F_: 290.7 Hz), 79.87, 74.28, 60.07, 54.05, 39.65, 36.61, 31.65, 30.39, 29.99, 29.92, 22.99 ppm; ESI-HRMS (C_14_H_23_N_2_O_5_): [M+H]^+^ *m/z*: 299.16019 (calcd. 299.16013).

**Scheme 2:**
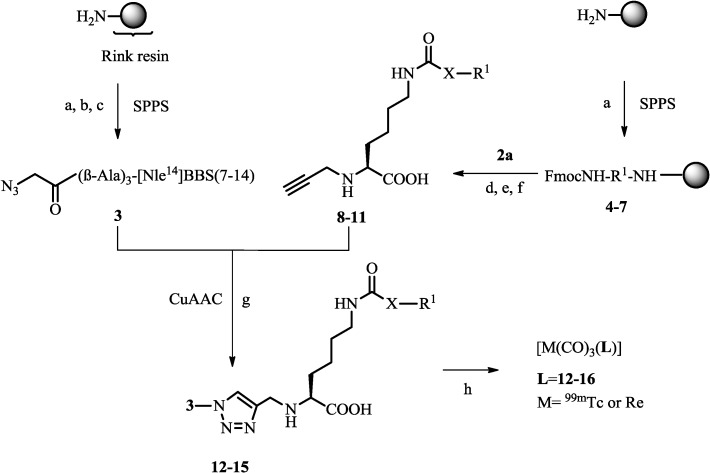
Synthesis of peptides and assembly of (radio)metal-labeled, multifunctional conjugates.

Solid-phase peptide synthesis (SPPS; scale: 0.03–0.25 mmol) was performed as described earlier [10,27]. Side-chain protected Fmoc-amino acids were Cys(Trt), Gln(Trt), His(Trt), Lys(Boc), Ser(tBu), and Trp(Boc). Azide-functionalized bombesin derivative **3** was synthesized according to the literature [10]. The different protected shepherdin peptide sequences [21,24] obtained by SPPS (**4**–**7**; 0.03–0.06 mmol) were subsequently coupled manually *via* selective amide bond formation to the central lysine precursor **2a** on solid support in the presence of HATU (2–3 equiv.) and *i*-Pr_2_NEt (5–6 equiv.) in DMF for 2 h at rt. After the coupling was completed, removal of protecting groups and cleavage of the peptides were done with a solution of trifluoroacetic acid, phenol, water, and triisopropylsilane (0.5–1 mL; 87.5/5/5/2.5%) at rt for 2–8 h. The precipitated crude peptides were dissolved in water, purified by preparative HPLC, and lyophilized. Afterwards, selective methyl ester hydrolysis of intermediates was achieved with LiOH (0.5 M; 1–3 h, rt) following a procedure described by Reddy *et al.* [29]. After reactions were completed and the solutions were neutralized with HCl (0.5 M), the crude peptides **8**–**11** were obtained (see Scheme 2). Finally, the alkyne functionalized shepherdin derivatives **8**–**11** were reacted with the purified azidoacetic acid-functionalized bombesin sequence **3** according to published procedures [30] *via* the Cu(I)-catalyzed alkyne-azide cycloaddition (CuAAC) [31,32] in solution. In brief, stoichiometric amounts of alkyne-shepherdin peptides **8**–**11** and azido-BBS peptide **3** (2 µmol) were dissolved in DMSO (400 µL) under an argon atmosphere and a freshly prepared Cu(I) solution was added (60 µL; 3 equiv.), prepared by mixing a CuSO_4_ pentahydrate solution (0.2 M, 30 µL) with an sodium ascorbate solution (0.4 M, 30 µL) on ice. The reaction was allowed to stir for 1 h at rt and completion of the reaction was checked by analytical HPLC. The click products were purified by preparative HPLC and lyophilized to obtain the final peptide conjugates **12**–**15** (see Table 1 and Scheme 2). Reference compound **16** (see Scheme 2) was synthesized as described earlier [10].

(a) SPPS: (i) piperidine in DMF; (ii) Fmoc-amino acids, TBTU-HOBt, (b) azidoacetic acid, HATU; (c) cleavage solution: trifluoroacetic acid, phenol, water, triisopropylsilane (87.5/5/5/2.5%), 2–5 h, rt; (d) SPPS: **2a**, HATU, (e) cleavage solution, 2–8 h, rt; (f) LiOH (0.5 M), 1–3 h, rt; (g) CuAAC in solution; CuSO_4_ (0.2 M), Na-ascorbate (0.4 M), 1 h, rt; (h) (radio)metal labeling; for M=^99m^Tc: **12**–**16** (0.1 mM, aq.), [^99m^Tc(CO)_3_(H_2_O)_3_]^+^, 100 °C, 30 min; for Re complexes: **12**–**16** (0.4 mM, aq.), [Et_4_N]_2_[Re(CO)_3_(Br)_3_], 100 °C, 60 min; AA = amino acid.

**Table:** 

compound (*:protected AA)	X	R^1^	
4*,8,12	-CH2CH2CO-	KHSSGCAFL	shepherdin [79-87]
5*,9,13	-CH2CH2CO-	SKLACFSHG	scrambled shepherdin [79-87]
6*,10,14	-CH2CH2CO-	KHSSG	shepherdin [79-83]
7*,11,15	-CH2CH2CO-	SGKHS	scrambled shepherdin [79-83]
**16**	-	CH3	reference compound

**Table 1:** Analytical data and yields of synthesized peptides and ^nat^Re-complexes thereof.

Compound	MALDI-MS; *m/z* (observed)	Yield [%]	Purity [%] ^c^
12	[M+H]^+^: 2432.23 (calcd.: 2432.24)	31 ^a^	92.9
13	[M+H]^+^: 2432.18 (calcd.: 2432.24)	10 ^a^	95.3
14	[M+H]^+^: 1998.01 (calcd.: 1998.04)	17 ^a^	98.3
15	[M+H]^+^: 1998.03 (calcd.: 1998.04)	18 ^a^	97.5
16	[M+H]^+^: 1458.75 (calcd.: 1458.80)	23 ^a^	97.6
[Re(CO)_3_(12)]	[M+H]^+^: 2700.10 (calcd.: 2700.17)	quant. ^b^	>98
[Re(CO)_3_(13)]	[M+H]^+^: 2700.16 (calcd.: 2700.17)	quant. ^b^	>98
[Re(CO)_3_(14)]	[M+H]^+^: 2265.95 (calcd.: 2265.97)	quant. ^b^	>98
[Re(CO)_3_(15)]	[M+H]^+^: 2265.93 (calcd.: 2265.97)	quant. ^b^	>98
[Re(CO)_3_(16)]	[M+H]^+^: 1712.7 (calcd.: 1712.71)	quant. ^b^	>98

^a^ overall yield of isolated conjugates; ^b^ conversion of starting material (HPLC); ^c^ determined by HPLC; for reference compound **16** see reference [10].

#### 2.2.2. (Radio)Metal Labeling of Peptides

Na[^99m^TcO_4_] was eluted from a Mallinckrodt ^99^Mo/^99m^Tc generator, and the precursor [^99m^Tc(CO)_3_(H_2_O)_3_]^+^ was prepared by adding [^99m^TcO_4_]^−^ (1.5–2 GBq) to the “CRS Isolink kit for tricarbonyl” and heating for 30 min at 100 °C. Aliquots of 1 mM stock solutions of peptides **12**–**16** in water (10 µL, 10 nmol) were added to a solution of [^99m^Tc(CO)_3_(H_2_O)_3_]^+^ (~100 MBq; 0.1 mM final peptide concentration) and the reaction mixtures were heated at 100 °C for 30 min. Quality controls were performed by radio-HPLC to determine the radiochemical yield and purity of [^99m^Tc(CO)_3_(**L**)] (L = **12**–**16**). For characterization purposes, the peptides were labeled with cold ^nat^Re. In brief, aliquots of aqueous stock solutions of peptides **12**–**16** (30–100 µL, 1 mM) were added to a solution of [Et_4_N]_2_[Re(CO)_3_(Br)_3_] [33] (50–150 µL, 1 mM) and heated for one hour at 100 °C [10]. The rhenium-complexes were purified by HPLC and analyzed by MS (see Table 1).

### 2.3. LogD Determinations

The hydrophilicity of the radiolabeled peptides **12**–**16** was evaluated by determination of their partition coefficient between *n*-octanol and phosphate buffered saline (PBS, pH 7.4) using the shake flask method [27]. In brief, 10 µL (1 µM) of radiolabeled peptides **12**–**16** were added to a pre-saturated solution of *n*-octanol and PBS (1:1, 1 mL) and the tubes were vortexed (1 min) and centrifuged (3,000 rpm, 10 min). Aliquots of 100 µL of each phase were radiometrically measured in a gamma counter and the logD values were calculated by the logarithm of the ratio between the radioactive counts in the octanol fraction and the radioactive counts in the PBS fraction. The experiment was performed 2–3 times, each in quintets, and results are reported by mean values ± standard deviation (SD).

### 2.4. In Vitro Experiments

Cell culturing of human Caucasian prostate adenocarcinoma (PC-3) cells and *in vitro* assays (internalization, receptor saturation binding, and externalization) were performed as previously described [10,27] and are thus described only in brief.

#### 2.4.1. Internalization Assay

For the determination of cellular uptake, PC-3 cells were incubated with the radiolabeled peptides [^99m^Tc(CO)_3_(**L**)] (**L** = **14**–**16**; 0.25 pmol; 1.5–2.0 kBq/well) for different time points to allow binding and internalization. Non-specific receptor binding and internalization was determined by incubating the cells with excess of natural bombesin(1–14) as a receptor blocking agent. At each time point, the supernatant was collected, representing the free radiopeptide fraction. The cell surface receptor bound fraction was obtained by treating the cells with an acidic saline glycine buffer (100 mM NaCl, 50 mM glycine, pH 2.8; 2 times for 5 min, on ice). The internalized fraction was determined by cell lysis with 1 M NaOH (10 min). Fractions of free, receptor-bound, and internalized radiopeptide were radiometrically measured in the gamma counter and calculated as percentage of applied dose normalized to 10^6^ cells for all time points (*n* = 2–3 in triplicates, reported by means ± SD) [10]. To verify whether specific uptake was influenced by the shepherdin sequence, internalization experiments were also conducted with excess shepherdin(79–87) (AnaSpec Inc., Fremont, CA, USA) as a blocking agent.

#### 2.4.2. Receptor Saturation Binding Assay

PC-3 cells were incubated with increasing concentrations (0.1–100 nM/well) of the radiolabeled peptides [^99m^Tc(CO)_3_(**L**)] (L = **14**–**16**) at 4 °C for 2 h. Non-specific binding was determined in the presence of excess of natural bombesin(1–14) as described above. Fractions of free and receptor-bound radioconjugates were radiometrically measured in the gamma counter for quantification and apparent binding dissociation constants (*K*_d, *app*_) were calculated (*n* = 2–4 in triplicates, reported by means ± SD) [10].

#### 2.4.3. Externalization Assay

PC-3 cells were incubated with the radiolabeled peptides [^99m^Tc(CO)_3_(**L**)] (**L** = **14**–**16**; 2.5 pmol; 14–21 kBq/well) for one hour to allow cell internalization. The free and receptor-bound radiopeptide fractions were removed as described above, fresh cell culture medium was added, and the cells were incubated for different time points (10–300 min). At each time-point, the externalized fractions were collected and the medium replaced. The remaining internalized amount of radioactivity was recovered by cell lysis. All fractions were radiometrically measured and calculated as percentage of the total internalized fraction (*n* = 4–7 in triplicates, reported by means ± SD) [10].

## 3. Results and Discussion

The syntheses of building blocks and multifunctional conjugates are depicted in Scheme 1 and Scheme 2. *N*(α)Boc-*N*(α)propargyl-Lys(OMe) (**1**) was synthesized from commercial BocLysOMe in five steps according to previously published procedures [28]. Reaction of compound **1** with succinic anhydride provided intermediate **2a** appropriately functionalized with a carboxylic acid functionality for conjugation reactions *via* amide bond formation. Shepherdin derivatives **4**–**7** [21,24] were synthesized by automated solid-phase peptide synthesis (SPPS) using Fmoc-chemistry. Compounds **5** and **7** with a scrambled amino acid sequences were prepared for control experiments. Peptides **4**–**7** were coupled to the Lys precursor **2a** on solid support, cleaved from the resin, and fully deprotected under standard conditions. Subsequent hydrolyses of the methyl ester of intermediates with LiOH provided alkyne-derivatized peptides **8**–**11**. The corresponding CuAAC reaction partner, N-terminally functionalized azido-(βAla)_3_[Nle^14^]BBS(7–14) **3**, was obtained by similar SPPS procedures [10]. A (βAla)_3_ spacer was introduced at the N-terminus of peptide **3** in order to prevent potential interference between the different moieties of the final multifunctional conjugate [9,10]. Reaction of alkyne-bearing shepherdin derivatives **8**–**11** and azido-BBS **3** by CuAAC in solution afforded final peptide conjugates **12**–**15**. For a side-by-side comparison, reference conjugate **16**, identical in all respects but lacking a shepherdin moiety (replaced by a methyl group; Scheme 2) was used, the synthesis and characterization of which has been previously described [10]. For analytical data of products see Table 1.

Radiolabeling of the triazole-containing peptide conjugates with ^99m^Tc-tricarbonyl was achieved following established procedures reported by us and others [10,28,34]. In brief, heating of aqueous solutions of 1,2,3-triazole containing peptide conjugates **12**–**16** with [^99m^Tc(CO)_3_(H_2_O)_3_]^+^ yielded the corresponding radiolabeled compounds [^99m^Tc(CO)_3_(**L**)] (**L** = **12**–**16**) in >95% radiochemical yield and purity and with a specific activity of up to 100 GBq/µmol. As common practice for the identification and characterization of n.c.a. (no carrier added) ^99m^Tc-labeled compounds, the corresponding non-radioactive analogous compounds [^nat^Re(CO)_3_(**L**)] (**L** = **12**–**16**) were also prepared by reaction of conjugates **12**–**16** with [Et_4_N]_2_[Re(CO)_3_(Br)_3_] [33] (for MS data see Table 1). Direct comparison of the UV-HPLC traces of the rhenium tricarbonyl complexes with the corresponding γ-HPLC traces of ^99m^Tc-tricarbonyl complexes confirmed in each case their identity (see Supplementary Information).

Already at this stage we recognized a high and persistent unspecific binding of conjugate [^99m^Tc(CO)_3_(**12**)] containing the full length shepherdin(79–87) sequence to material used as well as cell surfaces. In particular, the unspecific binding of [^99m^Tc(CO)_3_(**12**)] impeded with *in vitro* assays regardless of precautions taken (e.g., using prelubricated disposables, the addition of solvents (e.g., DMSO or EtOH), or varying the composition and ionic strength of the media; data not shown). We therefore focused henceforth on radioconjugates containing the truncated shepherdin(79–83) sequence for which unspecific binding was not an issue. Thus, the physico-chemical properties of compounds [^99m^Tc(CO)_3_(**L**)] (**L** = **14**,**15**) were evaluated in comparison to reference compound [^99m^Tc(CO)_3_(**16**)].

The lipophilicity of the conjugates was determined by the shake flask method. LogD values obtained ranged from −1.70 ± 0.13 and −1.68 ± 0.08 for compounds [^99m^Tc(CO)_3_(**14**)] and [^99m^Tc(CO)_3_(**15**)], respectively, which indicates an improved hydrophilicity in comparison to the reference compound [^99m^Tc(CO)_3_(**16**)] (logD = −0.44 ± 0.07).

**Figure 2:**
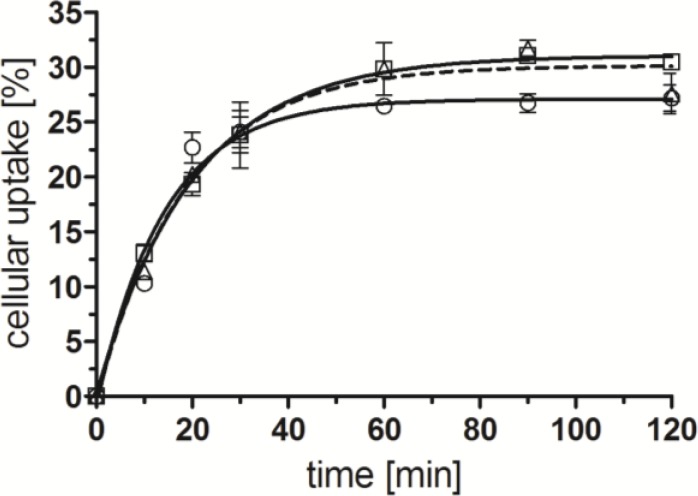
Receptor specific internalization of radiometal conjugates in PC-3 cells, overexpressing GRP-receptor; [^99m^Tc(CO)_3_(**14**)] (□, continuous line), [^99m^Tc(CO)_3_(**15**)] (∆, dotted line), [^99m^Tc(CO)_3_(**16**)] (○, continuous line); normalized to 10^6^ cells per well; *n* = 2–3 (in triplicates, reported by means ± SD, calculated by non-linear regression using GraphPad Prism 5.0).

BBS-shepherdin conjugate [^99m^Tc(CO)_3_(**14**)], its analog with the scrambled amino acid sequence [^99m^Tc(CO)_3_(**15**)], and reference compound [^99m^Tc(CO)_3_(**16**)] exhibited similar cell internalization profiles into GRP-r overexpressing PC-3 cells in terms of extent and rate of uptake (Figure 2). Approximately 25%–30% of the applied radioactivity was internalized within 30–60 min, which is comparable to related bombesin derivatives labeled with the ^99m^Tc-tricarbonyl core described by us and others [10,35]. Cell internalization was not influenced by the addition of shepherdin(79–87) and receptor-specific uptake was verified for all compounds by blocking experiments in the presence of excess natural bombesin(1-14) (data not shown).

**Figure 3:**
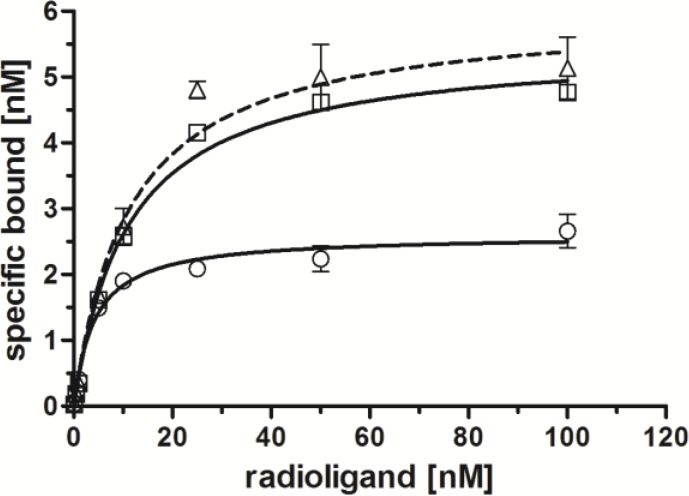
Specific receptor saturation binding to PC-3 cells; [^99m^Tc(CO)_3_(**14**)] (□, continuous line), [^99m^Tc(CO)_3_(**15**)] (∆, dotted line), [^99m^Tc(CO)_3_(**16**)] (○, continuous line); normalized to 10^6^ cells per well; *n* = 2–3 (in triplicates, reported by means ± SD, and calculated by non-linear regression using GraphPad Prism 5.0).

Binding affinities of radiolabeled conjugates [^99m^Tc(CO)_3_(**L**)] (**L** = **14**–**16**) towards the GRP-r were investigated by receptor binding saturation assays (Figure 3). All three derivatives showed high affinities towards GRP-r. Compared to reference compound [^99m^Tc(CO)_3_(**16**)] with an apparent dissociation constant (*K*_d, app_) of 5.6 ± 0.8 nM, dual-targeting BBS-shepherdin conjugate [^99m^Tc(CO)_3_(**14**)] revealed a slightly decreased receptor binding affinity (K_d, app_ = 10.9 ± 0.7 nM) as did its scrambled version [^99m^Tc(CO)_3_(**15**)] (*K*_d, app_ = 11.3 ± 2.2 nM). *K*_d_ values of receptor-specific radiopeptides in the low two-digit nanomolar range are still considered appropriate for applications *in vivo* [36,37].

**Figure 4:**
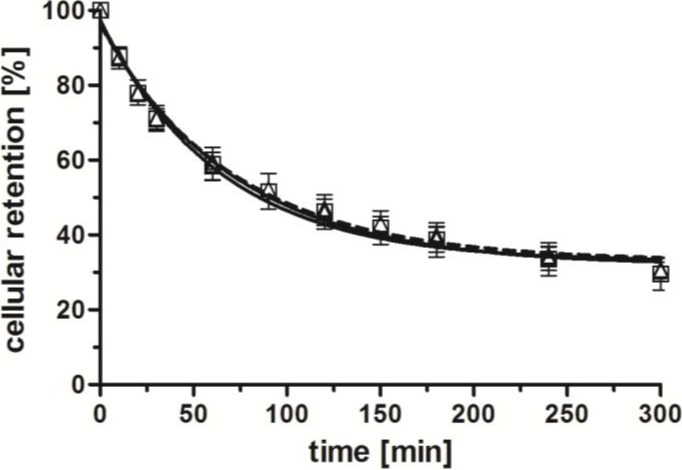
Externalization of radiolabeled compounds [^99m^Tc(CO)_3_(**L**)] (**L** = **14**–**16**) from PC-3 cells; [^99m^Tc(CO)_3_(**14**)] (□, continuous line), [^99m^Tc(CO)_3_(**15**)] (∆, dotted line), [^99m^Tc(CO)_3_(**16**)] (○, continuous line); *n* = 4–7 (in triplicates, reported by means ± SD; calculated by non-linear regression using GraphPad Prism 5.0).

After having verified the specificity and affinity of the radioconjugates for their tumor-specific, extracellular target (GRP-r), we next set out to study the effect of the conjugated shepherdin moiety with regards to the cellular retention of radioactivity. Unexpectedly, the externalization profiles of the three radioconjugates [^99m^Tc(CO)_3_(**L**)] (**L** = **14**–**16**) were comparable (Figure 4). A washout of 50% of the initially internalized radioactivity was observed within approximately 90 min for all conjugates. Thus, the additional shepherdin moiety of [^99m^Tc(CO)_3_(**14**)] did not lead to an improved cellular retention of radioactivity in comparison to compounds [^99m^Tc(CO)_3_(**L**)] (**L** = **15**–**16**). These results were puzzling because of the reported high affinity (80 nM) and specificity of shepherdin(79–83) and peptide conjugates thereof towards Hsp90 [20,21], the expression of which in PC-3 cells was verified by western blot experiments (data not shown). A potential explanation for our findings might include the possibility of endosomal entrapment of the conjugate as a consequence of endocytotic cell internalization, which in turn would prevent interactions of [^99m^Tc(CO)_3_(**14**)] with its cytosolic target Hsp90 [12,15,38]. Alternatively, enzymatic degradation of the peptidic radioconjugate within the intracellular endosome-lysosome cascade could result in the destruction of the shepherdin moiety and thus, loss of its specificity towards Hsp90 [15,38,39]. In either case, the intracellular fate of receptor-specific (radio)conjugates after cell internalization by endocytosis is generally not well understood in detail. There are recent reports suggesting solutions to overcome issues of endosomal entrapment including examples of the application of biomimetic peptides [40], cleavable linkers [41,42], and synthetic polymers [15]. In addition, reported non-peptidic inhibitors of Hsp90 [43] could be employed in order to address potential issues of the stability of the Hsp90-specific peptide moieties described herein. To address the challenging goal of improving the cellular retention of radioactivity after receptor-specific delivery to cancer cells by peptidic radiotracers, future research efforts will be directed towards the combination of our conjugates with the strategies outlined above.

## 4. Conclusions

We herein report the synthesis and *in vitro* evaluation of bombesin-shepherdin conjugates radiolabeled with the ^99m^Tc-tricarbonyl core by the “click-to-chelate” strategy. The multifunctional radioconjugates were designed for a combined extra- and intracellular targeting in order to improve the cellular retention of radioactivity after its receptor-specific delivery to cancerous cells. While the specificity of the radioconjugates towards the extracellular target (GRP-r) could be confirmed, the cellular externalization of radioactivity was not improved. The combination of extra- and intracellular targeting entities in a multifunctional radioconjugate represents a novel and innovative approach with potential to improve the efficacy of radiotracers provided that issues such as endosomal entrapment and lysosomal degradation can be addressed.

## Supplementary Files

Supplementary File 1 Supplementary Information (PDF, 1293 KB)
