# Supplementary material for: A Bombesin-Shepherdin Radioconjugate Designed for Combined Extra- and Intracellular Targeting
Source: Pharmaceuticals (Basel). 2014 May 27;7(6):662–75. doi: 10.3390/ph7060662 (PMC4078514; doi:10.3390/ph7060662)
Supplement: Supplementary File 1 — Supplementary Information (PDF, 1293 KB) [file pharmaceuticals-07-00662-s001.pdf]

Electronic Supplementary Information Fischer *et al.*

Table of contents

NMR spectra

^{1}H-NMR of compound **2b** (MeOH-d_4; recorded after H/D exchange) Figure S1.
^{13}C-NMR of compound **2b** (MeOH-d_4; recorded after H/D exchange) Figure S2.

MS spectra

HRMS (ESI) of compound **2b** Figure S3.
MS (MALDI-TOF) of compound **12** Figure S4.
MS (MALDI-TOF) of compound [Re(CO)$_3$(**12**)] Figure S5.
MS (MALDI-TOF) of compound **13** Figure S6.
MS (MALDI-TOF) of compound [Re(CO)$_3$(**13**)] Figure S7.
MS (MALDI-TOF) of compound **14** Figure S8.
MS (MALDI-TOF) of compound [Re(CO)$_3$(**14**)] Figure S9.
MS (MALDI-TOF) of compound **15** Figure S10.
MS (MALDI-TOF) of compound [Re(CO)$_3$(**15**)] Figure S11.

HPLC chromatograms

HPLC chromatograms of compound **14**, [Re(CO)$_3$(**14**)], and [^{99m}Tc(CO)$_3$(**14**)] Figure S12.
HPLC chromatograms of compound **15**, [Re(CO)$_3$(**15**)], and [^{99m}Tc(CO)$_3$(**15**)] Figure S13.

Figure S1. ^{1}H-NMR of compound **2b** (MeOH-d4; recorded after H/D exchange).

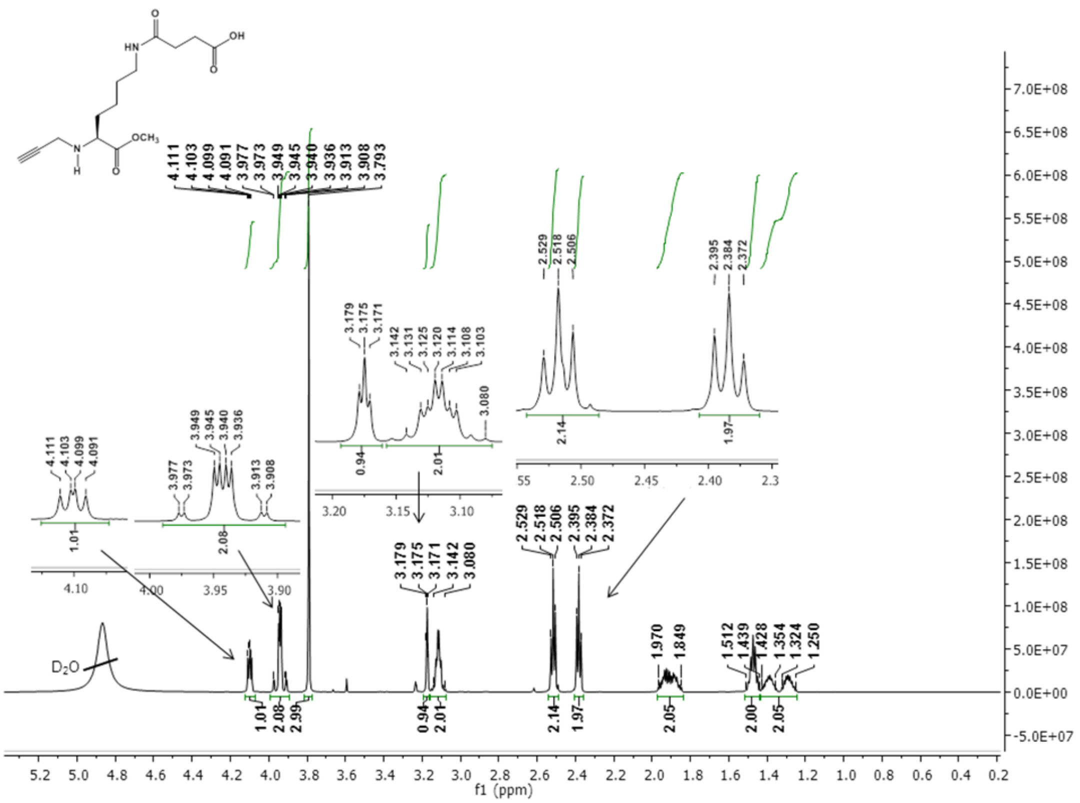

Figure S2. ^{13}C-NMR of compound **2b** (MeOH-d4; recorded after H/D exchange).

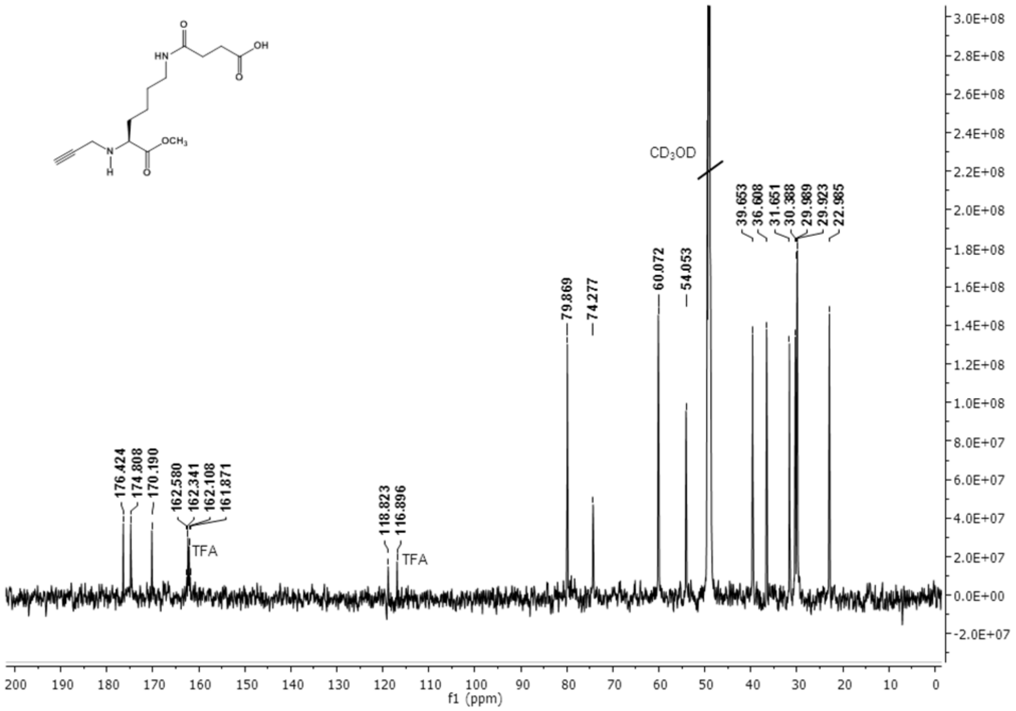

Figure S3. HRMS (ESI) of compound **2b**.

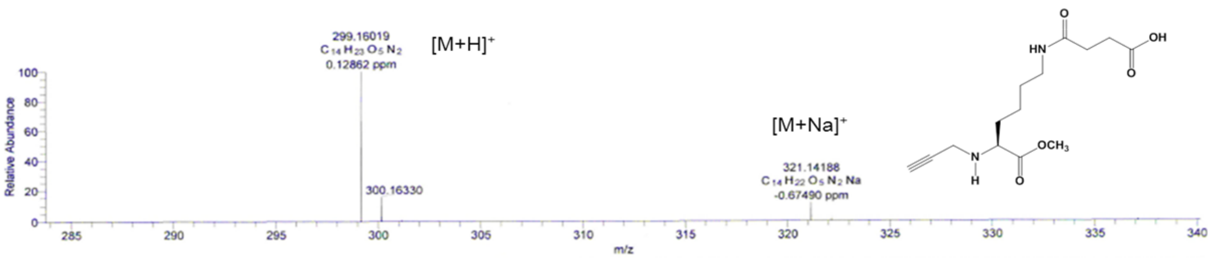

Figure S4. MS (MALDI-TOF) of compound **12**.

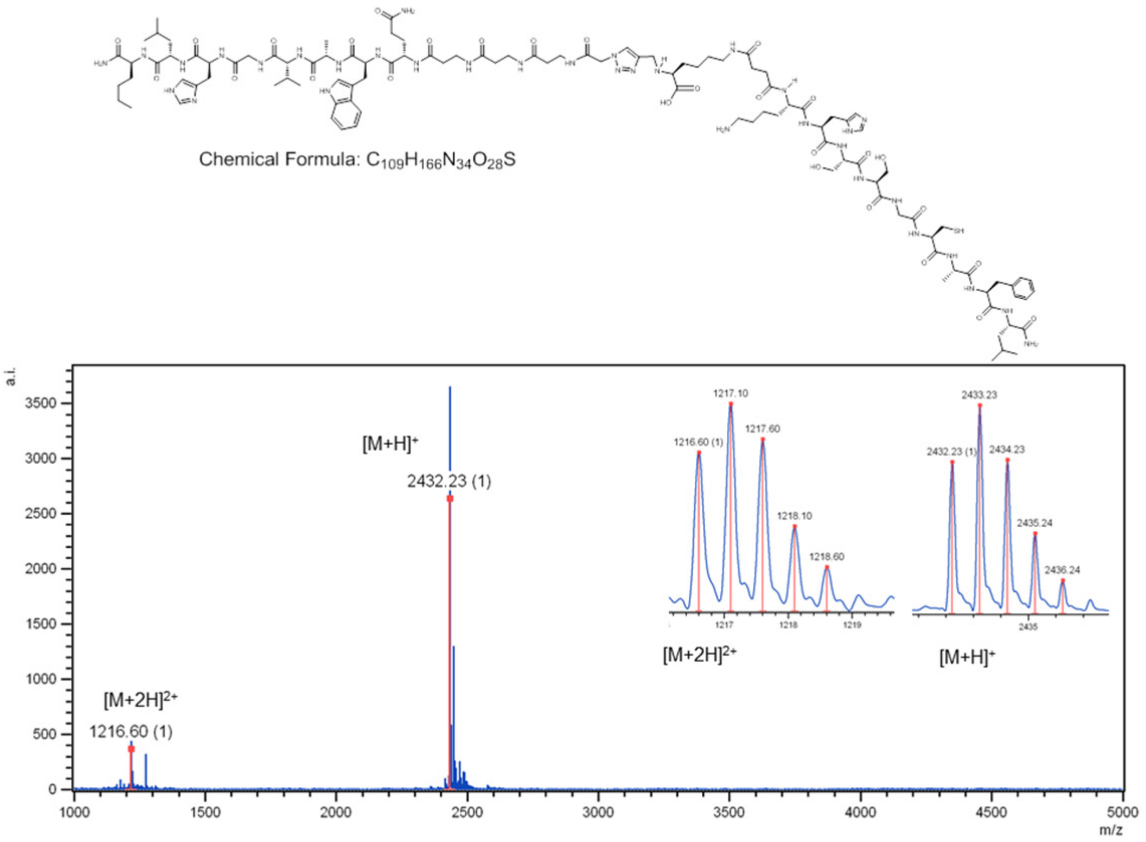

Figure S5. MS (MALDI-TOF) of compound [Re(CO)$_3$(**12**)].

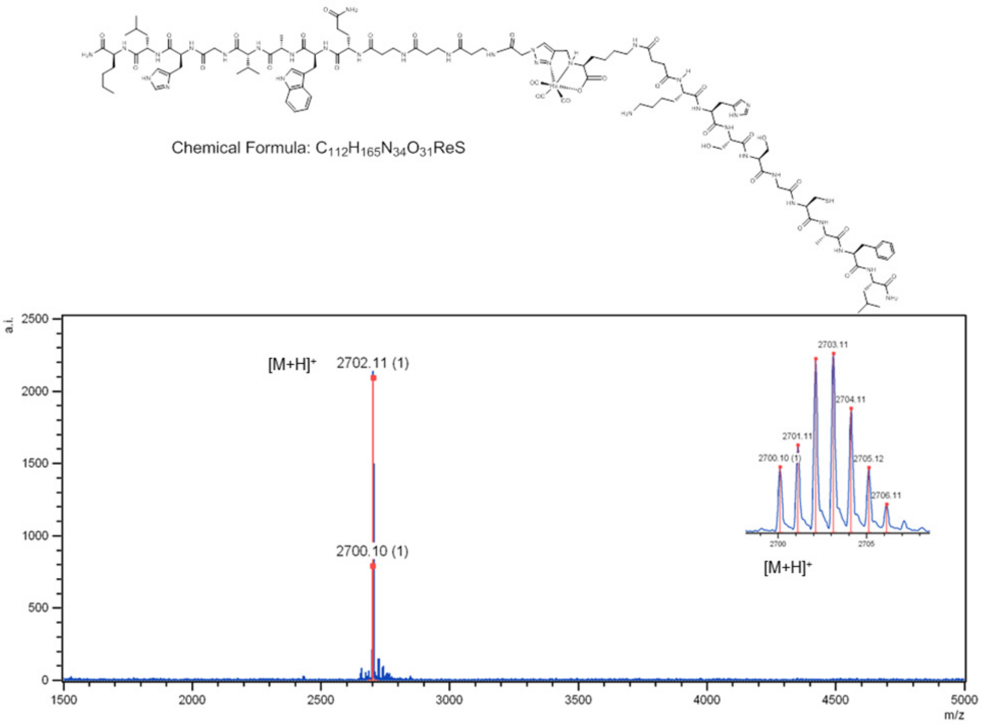

Figure S6. MS (MALDI-TOF) of compound **13**.

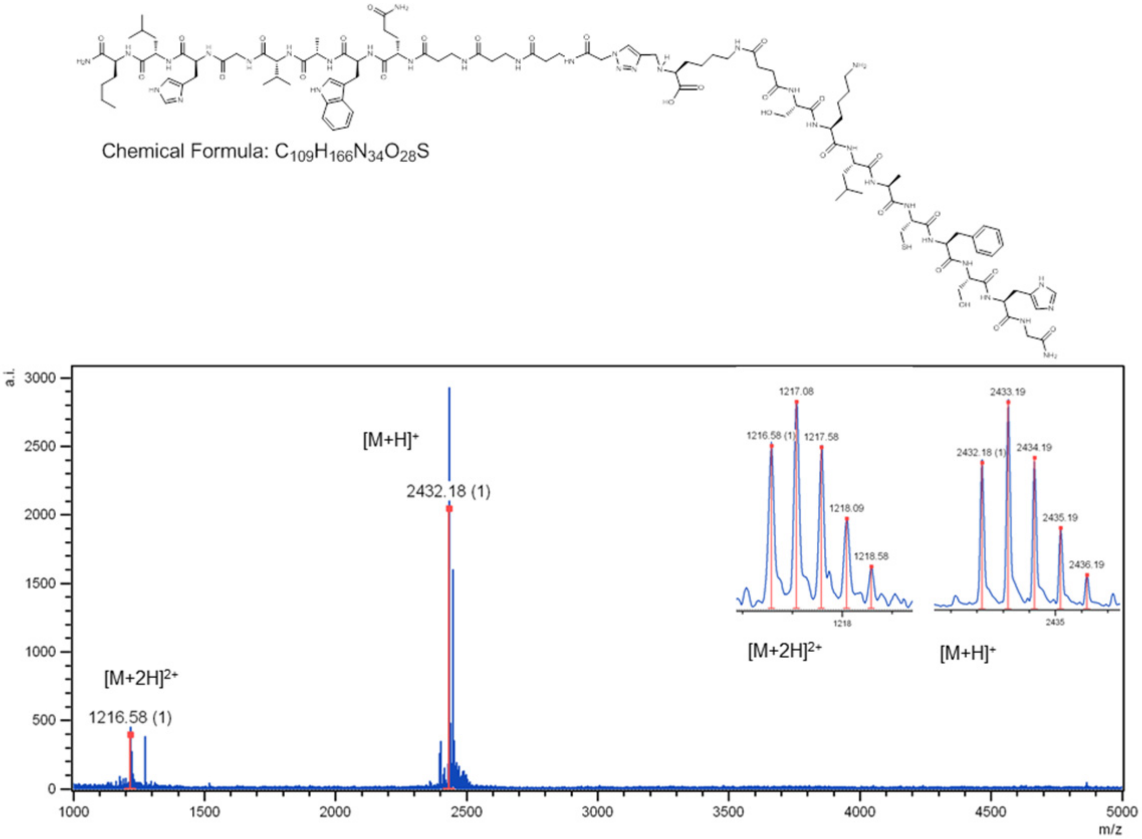

Figure S7. MS (MALDI-TOF) of compound [Re(CO)$_3$(**13**)].

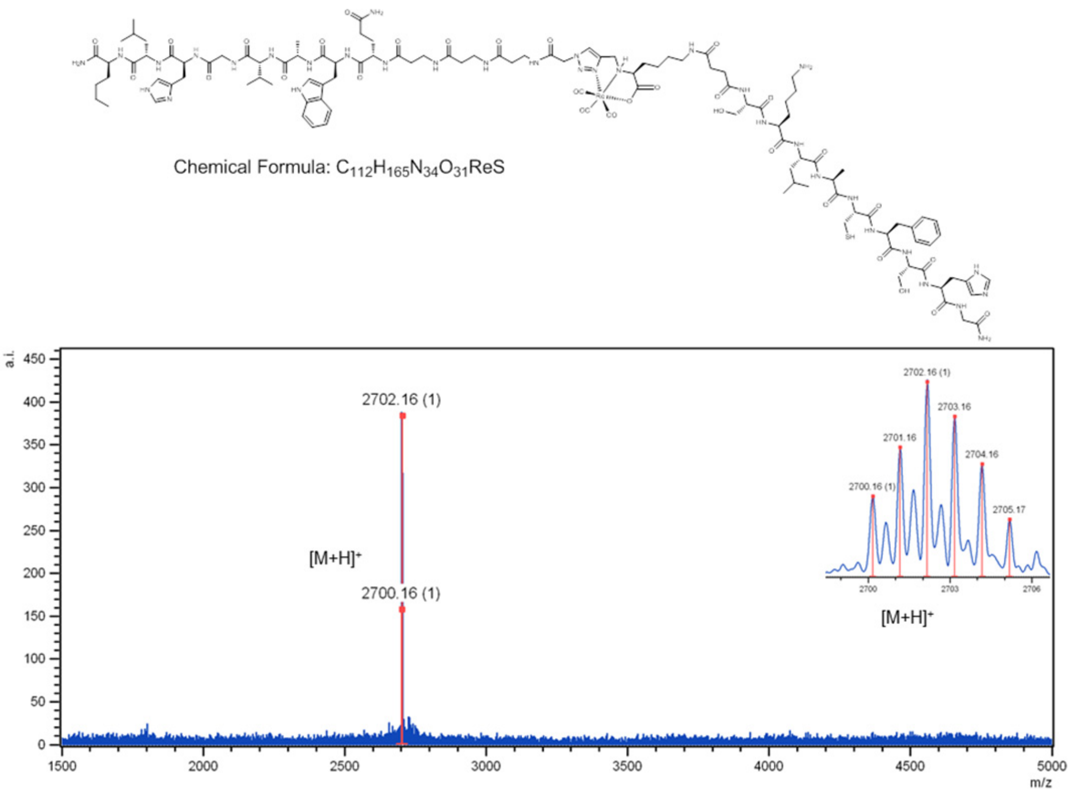

Figure S8. MS (MALDI-TOF) of compound **14**.

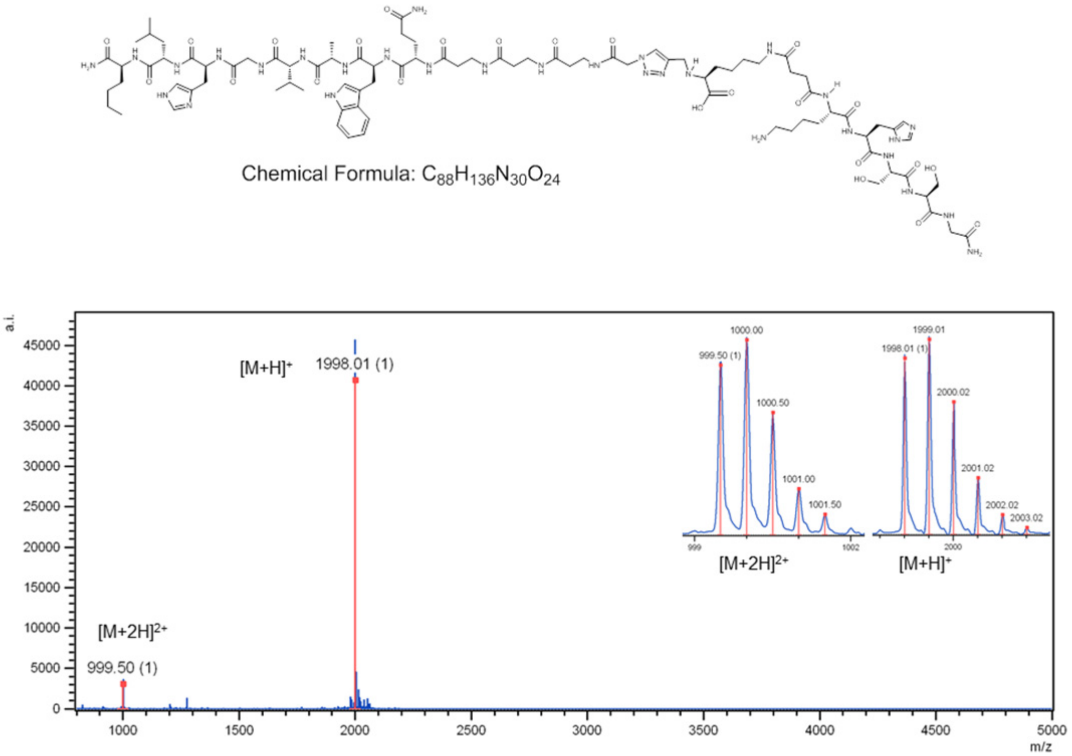

Figure S9. MS (MALDI-TOF) of compound [Re(CO)$_3$(**14**)].

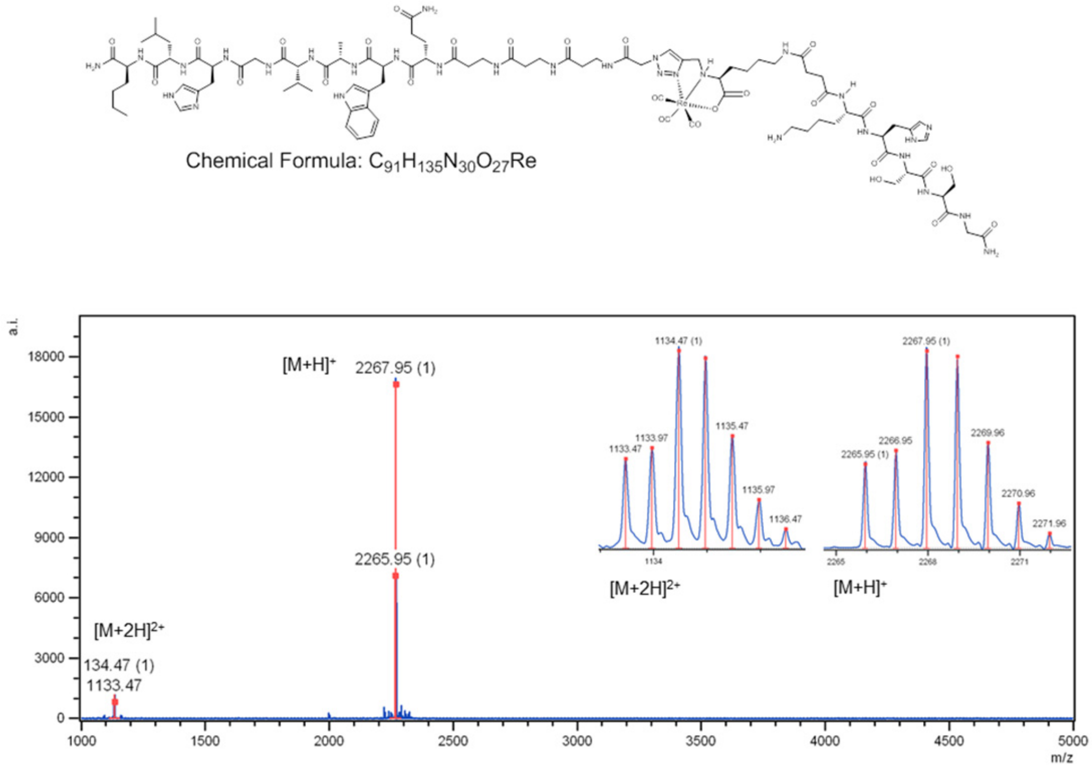

Figure S10. MS (MALDI-TOF) of compound **15.**

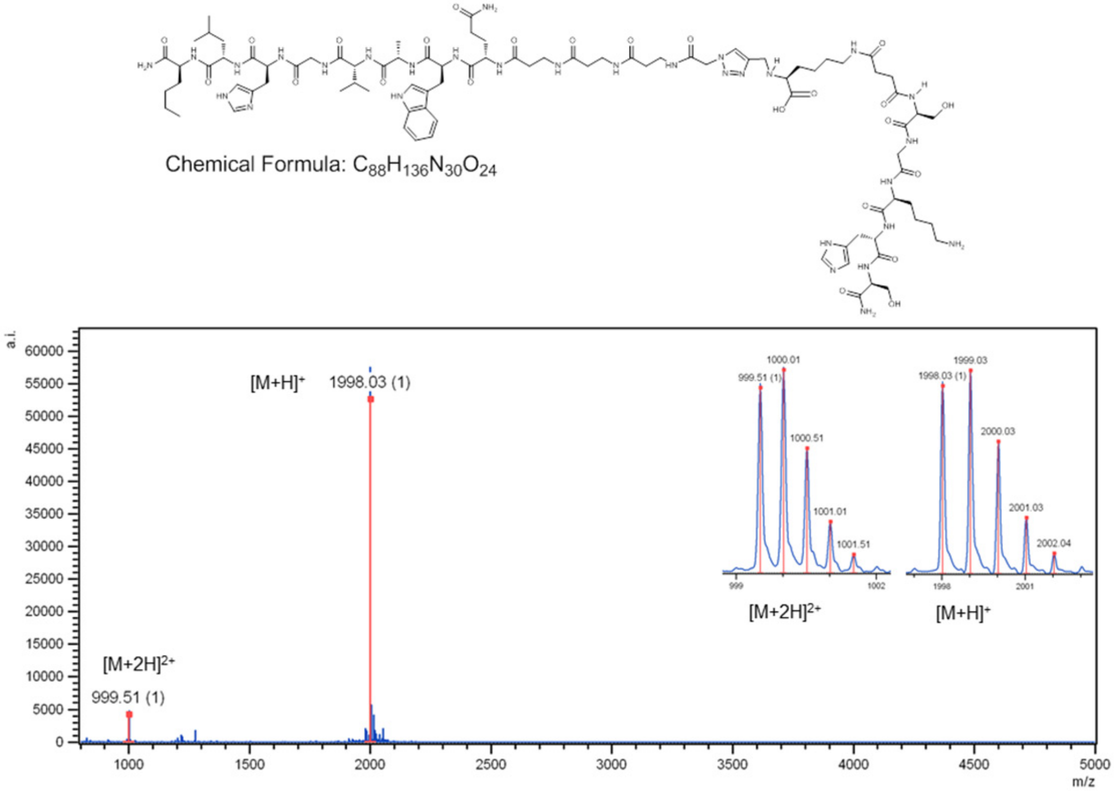

Figure S11. MS (MALDI-TOF) of compound [Re(CO)$_3$(**15**)].

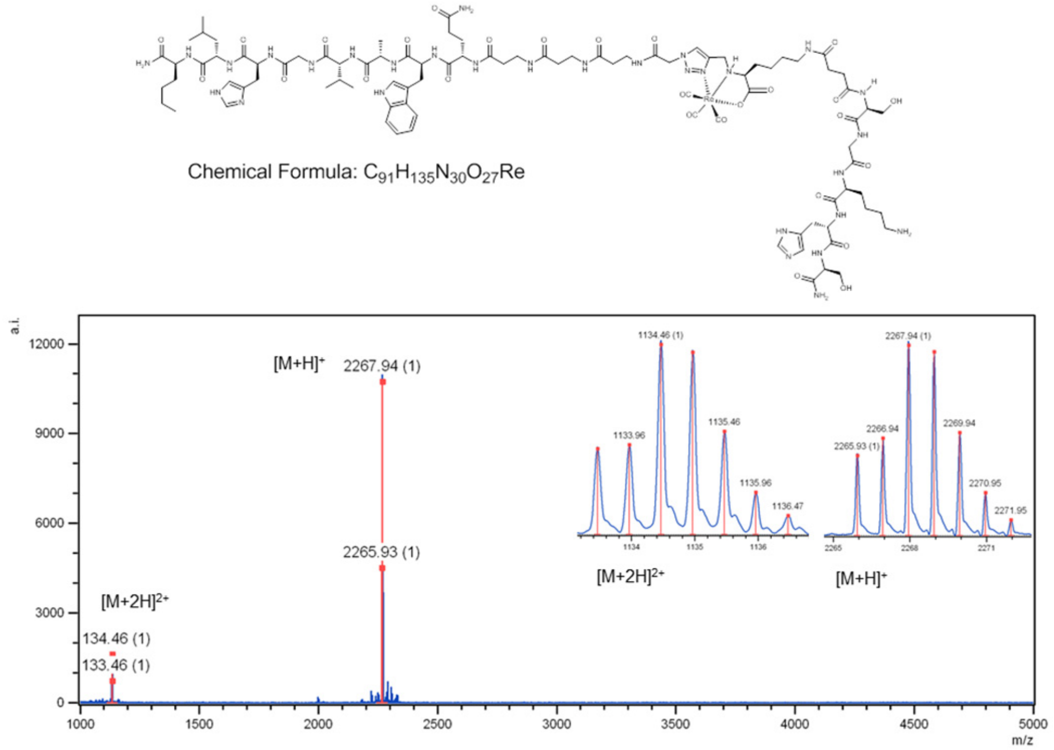

Figure S12. HPLC chromatograms of compound **14**, [Re(CO)$_3$(**14**)], and [^{99m}Tc(CO)$_3$(**14**)].

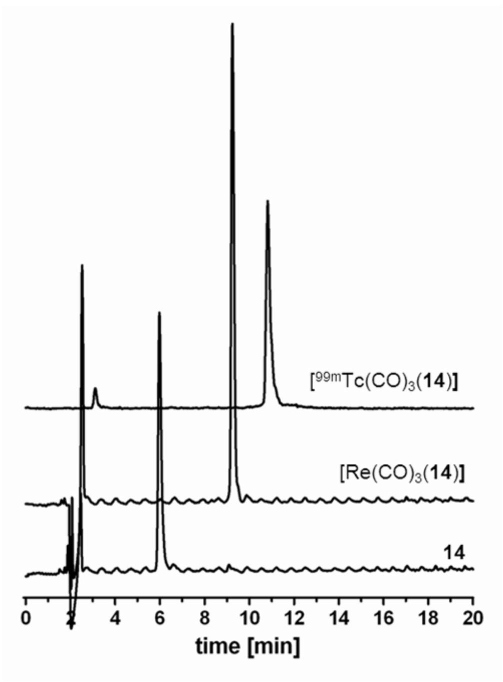

HPLC chromatograms of peptide **14** (UV-race, 214 nm), the corresponding metal conjugates [Re(CO)$_3$(**14**)] (UV-trace, 214 nm), and [^{99m}Tc(CO)$_3$(**14**)] (γ-trace); column A and a linear gradient from 80% A to 50 % A in 20 min with a flow rate of 1.5 mL/min. The small difference of retention times between [Re(CO)$_3$(**14**)] and [^{99m}Tc(CO)$_3$(**14**)] is due to the serial arrangement of the UV- and γ-detectors.

Figure S13. HPLC chromatograms of compound **15**, [Re(CO)$_3$(**15**)], and [^{99m}Tc(CO)$_3$(**15**)].

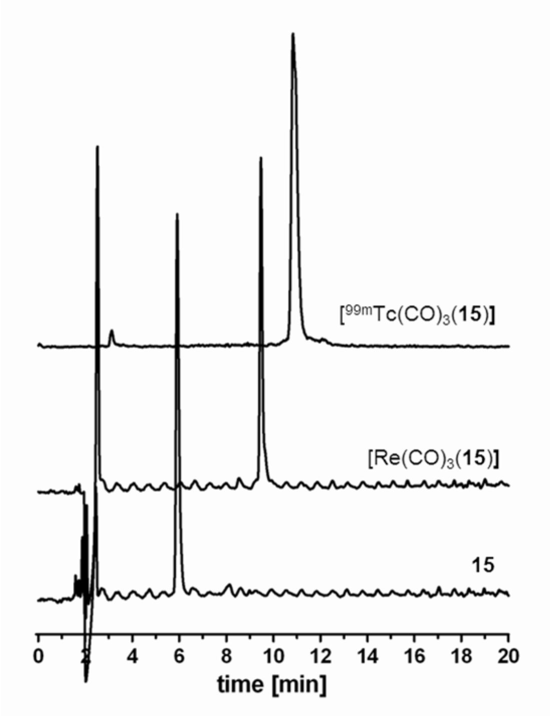

HPLC chromatograms of peptide **15** (UV-race, 214 nm), the corresponding metal conjugates [Re(CO)$_3$(**15**)] (UV-trace, 214 nm), and [^{99m}Tc(CO)$_3$(**15**)] (γ-trace); column A and a linear gradient from 80% A to 50 % A in 20 min with a flow rate of 1.5 mL/min. The small difference of retention times between [Re(CO)$_3$(**15**)] and [^{99m}Tc(CO)$_3$(**15**)] is due to the serial arrangement of the UV- and γ-detectors.
